# Supplementary material for: Targeted PARP Inhibition Combined with FGFR1 Blockade is Synthetically Lethal to Malignant Cells in Patients with Pancreatic Cancer
Source: Cells. 2020 Apr 8;9(4):911. doi: 10.3390/cells9040911 (PMC7226837; doi:10.3390/cells9040911)
Supplement: Supplementary file 1 [file cells-09-00911-s001.pdf]

SUPPLEMENTARY INFORMATION

**Targeted PARP inhibition in combination with FGFR1 blockade is synthetically lethal to malignant cells in patients with pancreatic cancer**

Shiue-Wei Lai[1,2,3], Oluwaseun Adebayo Bamodu[4,5], Jia-Hong Chen[1,2,3], Alexander TH Wu[6,7], Wei- Hwa Lee[5,8], Tsu-Yi Chao[1, 2,4,5,9*] and Chi-Tai Yeh[1, 2,4,5,10*]

[1] Graduate Institute of Clinical Medicine, Taipei Medical University, Taipei, Taiwan.

[2] Division of Hematology-Oncology, Department of Internal Medicine, Tri-Service General Hospital, National Defense Medical Center, Taipei, Taiwan.

[3] Department of Internal Medicine, Tri-Service General Hospital Penghu Branch, Penghu, Taiwan

[4] Department of Hematology and Oncology, Cancer Center, Taipei Medical University-Shuang Ho Hospital, New Taipei City, Taiwan

[5] Department of Medical Research & Education, Taipei Medical University-Shuang Ho Hospital, New Taipei City, Taiwan

[6] The PhD Program for Translational Medicine, College of Medical Science and Technology, Taipei Medical University and Academia Sinica, Taipei 110, Taiwan

[7] Graduate Institute of Medical Sciences, National Defense Medical Center, Taipei 114, Taiwan.

[8] Department of Pathology, Taipei Medical University-Shuang Ho Hospital, New Taipei City, Taiwan

[9]Taipei Cancer Center, Taipei Medical University, Taipei City 110, Taiwan

[10] Department of Medical Laboratory Science and Biotechnology, Yuanpei University of Medical Technology, Hsinchu City 30015, Taiwan

*Corresponding author(s):

Chi-Tai Yeh, PhD

Department of Medical Research and Education, Taipei Medical University - Shuang Ho Hospital, New Taipei City 23561, Taiwan. Tel: +886-2-2490088 ext. 8881, Fax: +886-2-2248-0900. E-mail: ctyeh@s.tmu.edu.tw

Tsu-Yi Chao, MD., PhD

Department of Hematology and Oncology, Cancer Center, Taipei Medical University - Shuang Ho Hospital, New Taipei City 23561, Taiwan. Tel: +886-2-2490088 ext. 8885, Fax: +886-2-2248-0900. E-mail: 10575@s.tmu.edu.tw

**Supplementary Table S1.** Western blot antibodies sheet.

| REAGENT or RESOURCE | SOURCE | IDENTIFIER (Cat. No.) |
|---|---|---|
| **Drugs** | | |
| PD 173074, >95% (HPLC), powder | Sigma-Aldrich | 219580-11-7 |
| Olaparib (AZD2281, Ku-0059436) | Selleck Chemicals | S1060 |
| **Antibodies** | | |
| Anti-Ki67 antibody | Abcam | ab15580 |
| Recombinant Anti-Bax antibody [E63] | Abcam | ab32503 |
| Recombinant Anti-Cleaved PARP1 antibody [E51] | Abcam | ab32064 |
| FGF Receptor 1 (D8E4) XP® Rabbit mAb | Cell Signaling | #9740 |
| PARP (46D11) Rabbit mAb | Cell Signaling | #9532 |
| Cleaved PARP (Asp214) (D64E10) XP® Rabbit mAb | Cell Signaling | #5625 |
| Caspase-9 (C9) Mouse mAb | Cell Signaling | #9508 |
| Caspase-3 (3G2) Mouse mAb | Cell Signaling | #9668 |
| Bcl-xL (54H6) Rabbit mAb | Cell Signaling | #2764 |
| β-Actin Antibody (AC-15) | Santa Cruz | sc-69879 |

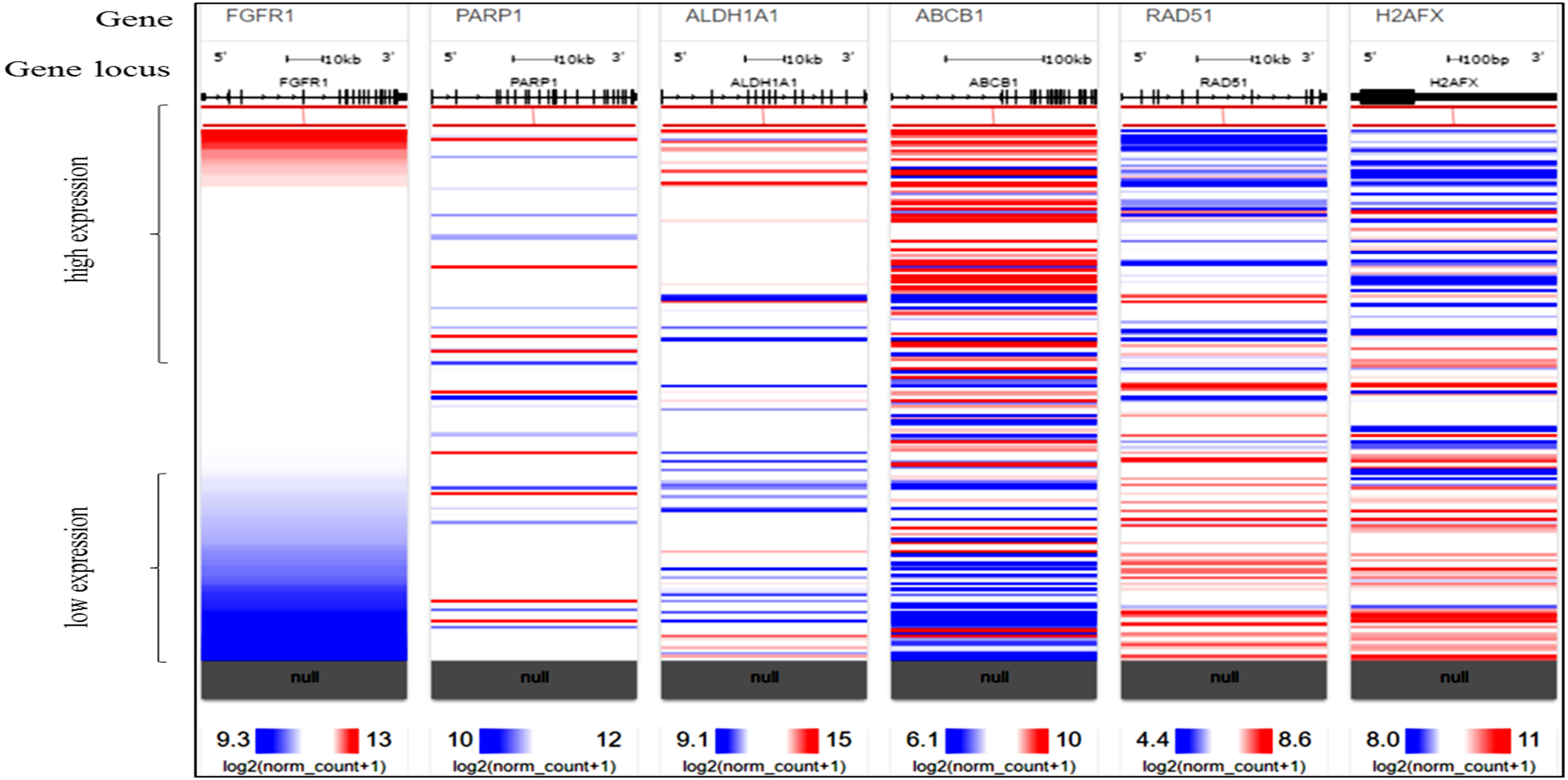


**Supplementary Figure S1.** Aberrant FGFR1 and PARP1 expression is associated with cancer stem cell-like phenotype, regulates DNA repair, and modulate response to therapy in PDAC cells.

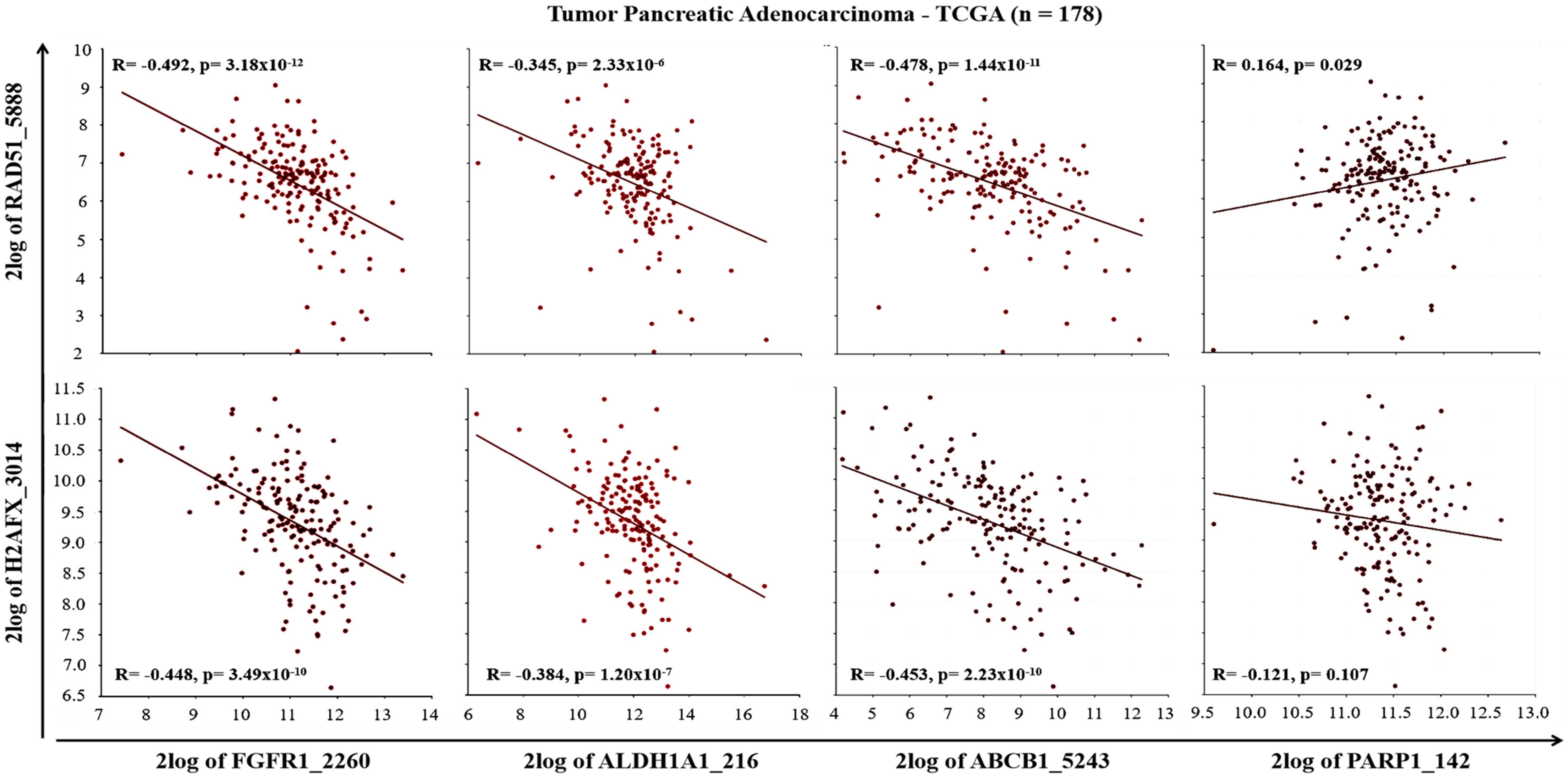


**Supplementary Figure S2.** Dots and line plots of the correlation between FGFR1, PARP1, ALDH1A1, ABCB1, RAD51 and H2AFX gene expression in the Tumore Pancreatic Adenocarcinoma (n = 178).

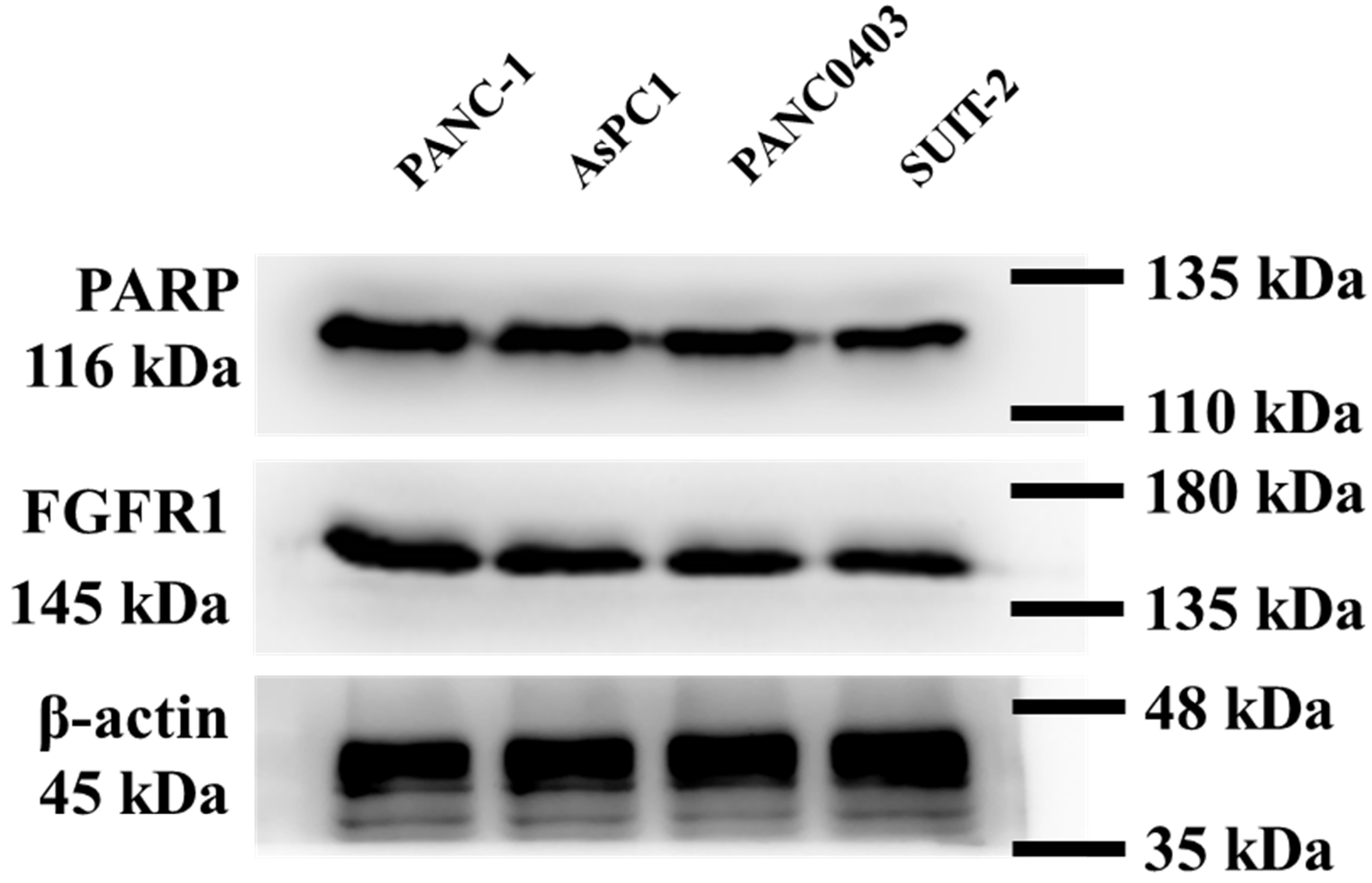


**Supplementary Figure S3.** Full-size blots of Figure 1B

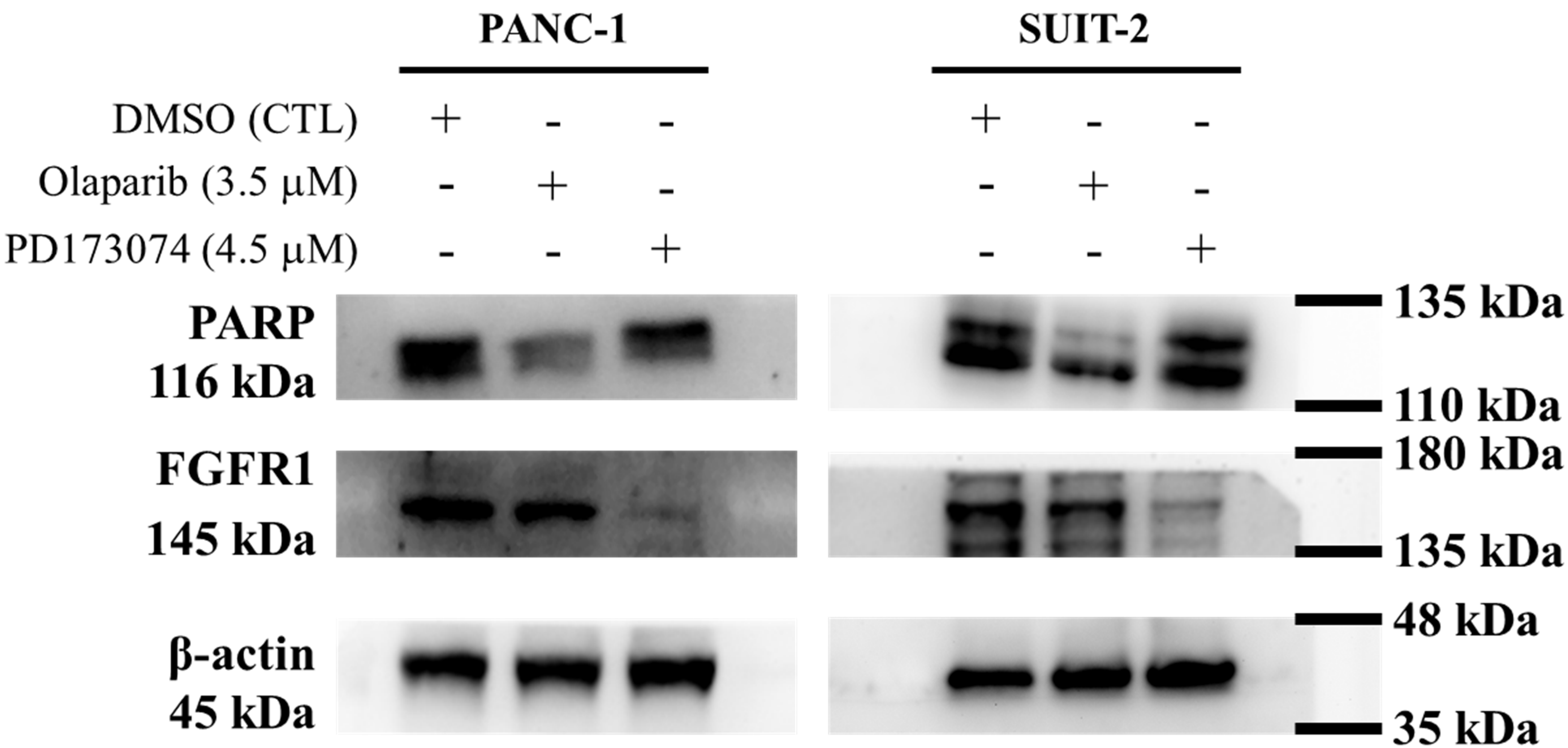


**Supplementary Figure S4.** Full-size blots of Figure 1D

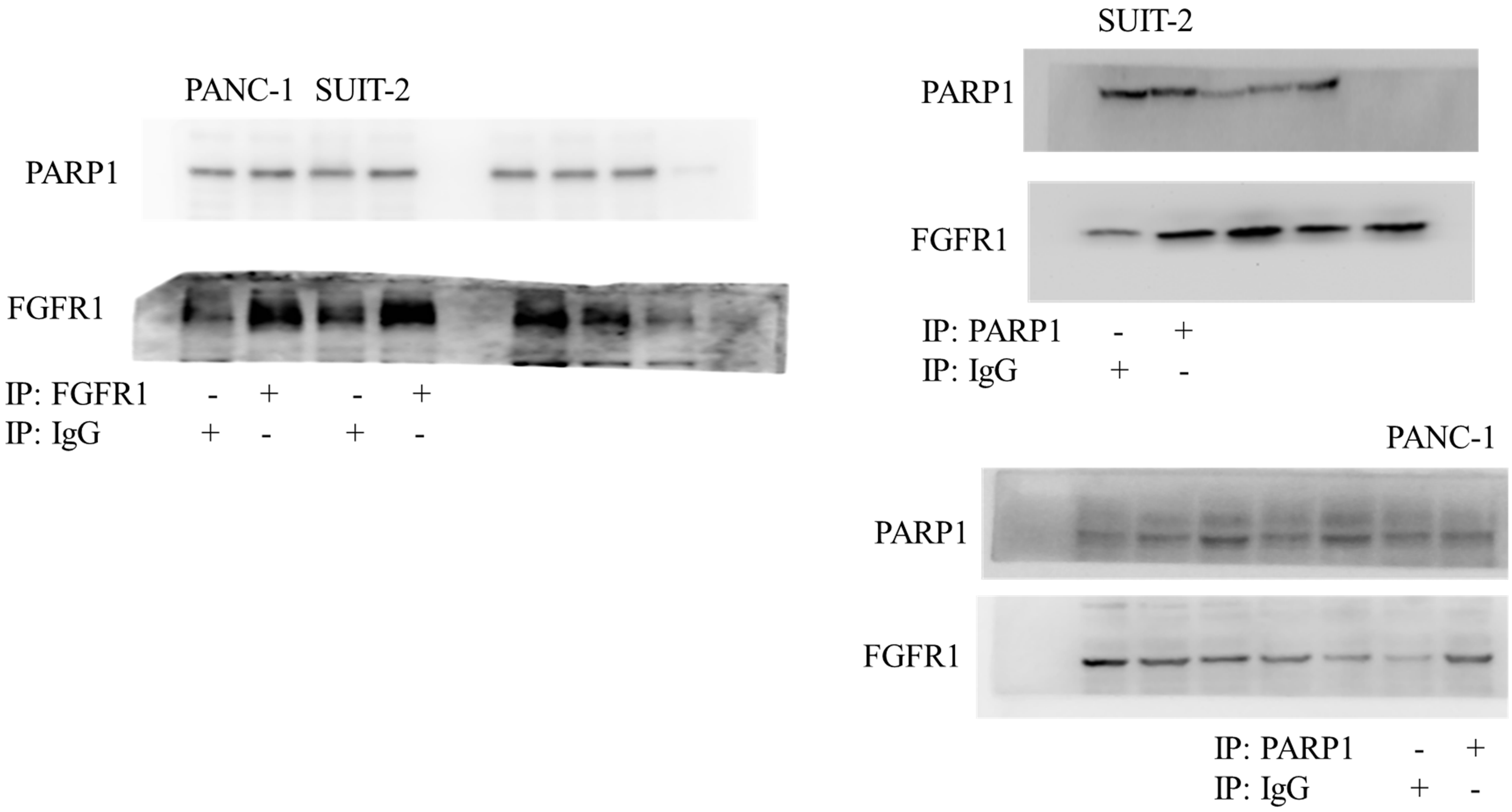


**Supplementary Figure S5.** Full-size blots of Figure 3D

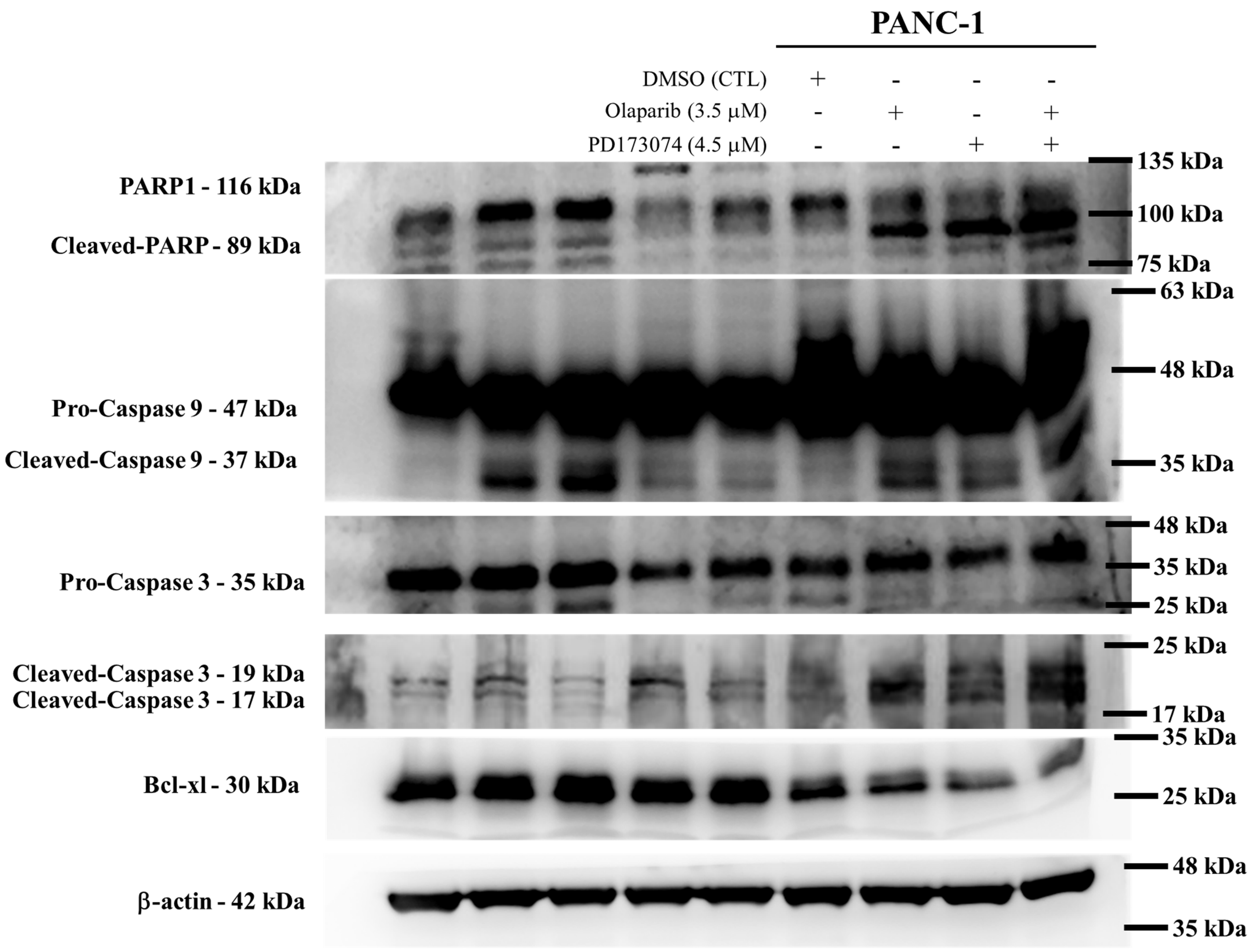


**Supplementary Figure S6.** Full-size blots of Figure 4D
